# Supplementary material for: MYB orchestrates T cell exhaustion and response to checkpoint inhibition
Source: Nature. 2022 Aug 17;609(7926):354–60. doi: 10.1038/s41586-022-05105-1 (PMC9452299; doi:10.1038/s41586-022-05105-1)
Supplement: Supplementary file 1 [file 41586_2022_5105_MOESM1_ESM.pdf]

Corresponding author(s): Axel Kallies
Veit R. Buchholz

Last updated by author(s): Dec 1, 2021

Reporting Summary

Nature Portfolio wishes to improve the reproducibility of the work that we publish. This form provides structure for consistency and transparency in reporting. For further information on Nature Portfolio policies, see our Editorial Policies and the Editorial Policy Checklist.

Statistics

For all statistical analyses, confirm that the following items are present in the figure legend, table legend, main text, or Methods section.

n/a	Confirmed	
☐	☒	The exact sample size (*n*) for each experimental group/condition, given as a discrete number and unit of measurement
☐	☒	A statement on whether measurements were taken from distinct samples or whether the same sample was measured repeatedly
☐	☒	The statistical test(s) used AND whether they are one- or two-sided<br>*Only common tests should be described solely by name; describe more complex techniques in the Methods section.*
☐	☒	A description of all covariates tested
☐	☒	A description of any assumptions or corrections, such as tests of normality and adjustment for multiple comparisons
☐	☒	A full description of the statistical parameters including central tendency (e.g. means) or other basic estimates (e.g. regression coefficient) AND variation (e.g. standard deviation) or associated estimates of uncertainty (e.g. confidence intervals)
☒	☐	For null hypothesis testing, the test statistic (e.g. *F*, *t*, *r*) with confidence intervals, effect sizes, degrees of freedom and *P* value noted<br>*Give P values as exact values whenever suitable.*
☒	☐	For Bayesian analysis, information on the choice of priors and Markov chain Monte Carlo settings
☒	☐	For hierarchical and complex designs, identification of the appropriate level for tests and full reporting of outcomes
☒	☐	Estimates of effect sizes (e.g. Cohen's *d*, Pearson's *r*), indicating how they were calculated

Our web collection on statistics for biologists contains articles on many of the points above.

Software and code

Policy information about availability of computer code

Data collection	Flow Cytometry:<br>CytExpert (Beckman Coulter) v2.3.1.22
Data analysis	Flow Cytometry:<br>FlowJo v10.6.1<br><br>Data analysis and visualization:<br>Prism v7<br><br>Visualization:<br>Adobe Illustrator v24.1.2<br><br>Annotation of scRNA-seq data:<br>CellRanger v5.0.0<br><br>scRNA-seq analysis:<br>SCANPY v1.6<br>Velocyto 0.17.17<br>scVelo v0.2.1

For manuscripts utilizing custom algorithms or software that are central to the research but not yet described in published literature, software must be made available to editors and reviewers. We strongly encourage code deposition in a community repository (e.g. GitHub). See the Nature Portfolio guidelines for submitting code & software for further information.

Data

Policy information about availability of data

All manuscripts must include a data availability statement. This statement should provide the following information, where applicable:

- Accession codes, unique identifiers, or web links for publicly available datasets
- A description of any restrictions on data availability
- For clinical datasets or third party data, please ensure that the statement adheres to our policy

Mathematical codes are available upon request.

Field-specific reporting

Please select the one below that is the best fit for your research. If you are not sure, read the appropriate sections before making your selection.

☒ Life sciences ☐ Behavioural & social sciences ☐ Ecological, evolutionary & environmental sciences

For a reference copy of the document with all sections, see nature.com/documents/nr-reporting-summary-flat.pdf

Life sciences study design

All studies must disclose on these points even when the disclosure is negative.

Sample size	No sample-size calculation was performed, the data presented in this study was collected in repeated independent experiments with at least 2-3 mice per group, as indicated in the Figure Legends.
Data exclusions	No data were excluded from the analysis.
Replication	The presented data was successfully replicated, in some cases experiments were replicated by different authors.
Randomization	Age- and sex-matched mice were allocated to groups based on the experimental treatment (no randomization).
Blinding	Blinding was not performed, as data analysis was strictly quantitative and not subjective. Computational analysis was not blinded.

Reporting for specific materials, systems and methods

We require information from authors about some types of materials, experimental systems and methods used in many studies. Here, indicate whether each material, system or method listed is relevant to your study. If you are not sure if a list item applies to your research, read the appropriate section before selecting a response.

Materials & experimental systems

n/a	Involved in the study	
☐	☒	Antibodies
☒	☐	Eukaryotic cell lines
☒	☐	Palaeontology and archaeology
☐	☒	Animals and other organisms
☒	☐	Human research participants
☒	☐	Clinical data
☒	☐	Dual use research of concern

Methods

n/a	Involved in the study	
☒	☐	ChIP-seq
☐	☒	Flow cytometry
☒	☐	MRI-based neuroimaging

Antibodies

Antibodies used

Name, Clone, Supplier:
CD45.1, A20, Biolegend or BD Bioscience
CD45.2, 104, BD Bioscience
CD90.1, HIS51, Thermo Fisher Scientific
CD4, RM4-5, Biolegend
CD4, GK1.5, Biolegend
CD44, IM7, BD Bioscience
CD19, 6D5, Biolegend
CD117, ACK2, Thermo Fisher Scientific
CD101, Moushi101, Thermo Fisher Scientific
CD160, eBioCNX46-3, eBioscience
CD244, eBio244F4, Thermo Fisher Scientific

CD90.2, 30-H12, Biolegend
CD69, H1.2F3, Biolegend
CD62L, MEL-14, Biolegend
CD8, 53-6.7, Biolegend or BD Bioscience
CX3CR1, SA011F11, Biolegend
Ki-67, 16A8, Biolegend
KLRG1, 2F1, Biolegend
Ly108, 330-AJ, Biolegend
PD1, 29F1.A12, Biolegend
PD1, RMPI-30, Biolegend
Tim3, RMT3-23, Biolegend
Tigit, GIGD7, Thermo Fisher Scientific
Tox, TXRX10, Thermo Fisher Scientific

Validation

All antibodies were obtained commercially and validation was based on the descriptions provided on the manufacturer's homepage.

Animals and other organisms

Policy information about studies involving animals; ARRIVE guidelines recommended for reporting animal research

Laboratory animals

- 6-8 weeks C57BL/6 mice were obtained from the Australian Resources Centre or Envigo
- P14 TCR transgenic mice expressing diverse combinations of the congenic markers CD45.1/.2 and CD90.1/.2, as well as TCRa knockout mice were bred at the mouse facility of Technische Universität München, München, Germany.
Age- and sex-matched mice (all on C57BL/6 background) were used in this study and entered experiments at 6-8 weeks of age.
- MybGFP mice and Mybfl/flCd4Cre mice were bred and housed at the mouse facility of Peter Doherty Institute of Infection and Immunity

Wild animals

The study did not involve wild animals.

Field-collected samples

The study did not involve samples collected in the field.

Ethics oversight

Experimental protocols were approved by the committee for experimentation with laboratory animals of the district government of Upper Bavaria (Germany) or University of Melbourne Animal Ethics Commitee.

Note that full information on the approval of the study protocol must also be provided in the manuscript.

Flow Cytometry

Plots

Confirm that:

☒ The axis labels state the marker and fluorochrome used (e.g. CD4-FITC).

☒ The axis scales are clearly visible. Include numbers along axes only for bottom left plot of group (a 'group' is an analysis of identical markers).

☒ All plots are contour plots with outliers or pseudocolor plots.

☒ A numerical value for number of cells or percentage (with statistics) is provided.

Methodology

Sample preparation

Single cell suspensions from different organs were obtained as described in the methods section.

Instrument

Cell sorting was performed on a MoFlo Astrios (Beckman Coulter). For data collection, a Cytoflex Lx cytometer (Beckman Coulter) was used.

Software

CytExpert (Beckman Coulter) v2.3.1.22 software was used for data collection. The acquired samples were analyzed using FlowJo software v10.6.1.

Cell population abundance

Sort purities were routinely confirmed, as assessed by post-sort measurements of the respective target cell populations.

Gating strategy

1. FSC/SSC gates were used to select lymphocyte populations.
2. FSC/FSC width gates were used to identify singlets.
3. Live/dead exclusion was performed using propidium iodide (PI) or eBioscience Fixable Viability Dye eF780
4. Further gating of target-cell populations relied on the respective marker combinations, for cell surface or intracellular markers, used in the specific experiments.

☒ Tick this box to confirm that a figure exemplifying the gating strategy is provided in the Supplementary Information.
